# Observations on the Treatment of Small-Pox

**Published:** 1829-07-01

**Authors:** 


### Some Observations on the Treatment of Small-pox. By Alex. Stewart, A.B.T.D. Assist. Surg. 2d Dragoons.

We lay before our readers the following extract from a printed paper which we received by the post. We suppose the article is in the course of publication in the Dublin Hospital Reports. We pass no opinion on the practice which Mr. Stewart recommends as preventive of deformity from small-pox.

"Whilst the pustule is yet Lymphic, (I would almost say papular) and before much, or any ulceration and suppuration has taken place, to pass a needle through it, as near the base as possible, and having a small bit of dry lint* in the other hand to press the

* A separate bit for each pustule.

apex gently on the base, and there retain it about a minute or two and then destroy the lint, this is to be done to all separately, and individually, as they appear: The effect on each is various —in some, an almost immediate cohesion will take place between the apex and the base, and a small superficial scab will be the consequence, the ulcerative action merges into adhesion, the red basis gradually subsides, and when in a few days, this superficial scab falls off, the part is healed without pitting—in others, the little pustule will again fill: puncture and press it down, the apex and base may then unite by the first intention, or it may again require a third time the operation, but seldom have I in any case known it to require more. The constitutional treatment must of course be adapted to the circumstances of the case, as if this mechanical and local one had not been made use of. It may be objected that it is laborious going over each pustule, when a full crop has covered the surface, but we must rejoin, that we cannot have any thing without trouble—maternal solicitude will not find it irksome —at first the motion requisite will greatly disturb the child and distress the parent, and perhaps render her unwilling to pursue the task, but the evident relief so shortly produced will raise her hopes, and render the process pleasant. When confluence is apprehended, and two or more pustules are so close that their inflamed bases are united, puncture each separately as far as possible from each other at the same time, and press between them with lint, the contained lymph is absorbed from each as it is pressed out, the adhesion of the apex and base is separately produced, and confluence is prevented. The rationale of the above practice must be evident to every person who will reflect, that febrile actions producing inflammatory affections of the skin, will be necessarily increased in a compound ratio of the specific tendency of that Inflammation, and the state of the system in which the disease has been produced; that if, as in Small Pox, the tendency is to run into suppurative and ulcerative inflammation, the general irritation consequent to such action must necessarily react considerably on the constitutional derangement, and augment the fever; the above method, then, by removing a cause, will also remove an effect, the primary being lessened, and the secondary fever either not occurring or with much mildness; in fact, the first intention is substituted for the second, and the disease strangled in its birth. The local consequence of this to females is invaluable, as little if any pitting is to be found after the part is healed—I am not aware of this method being in practice; pustules, vesicles, and papulæ, have been broken down in all the stages of the disease,* but it would appear to me, more to obtain fluid for experiment, or to see the nature of the contained fluid, than for a curative purpose, no pressure being used, nor the above mode of practice proposed. The idea suggested itself to me, when in the country, on the 21st of November, 1827. A man, not belonging to the regiment, requested me to attend his son, a fine boy about three years old, then covered thickly with eruption; and Small Pox prevalent in the neighbourhood, being aware that puncturing after maturation is sometimes recommended with a view to prevent the absorption of pus, many considering such circumstance an additional and reacting cause of febrile excitement, and also considering that ulcerative inflammation was the principal cause of the after pitting on the surface. It appeared to me, that early puncturing, and bringing the parts together before maturation, and while under a comparatively simple inflammatory excitement, a new and healthy action might be produced, and the specific tendency to suppuration and its consequences destroyed—the event justified the opinion. Mr. John Hunter has demonstrated by dissection, that a slough exists in the cutis in Small Pox, answering to the size of the pock, and which he considers peculiar to that disease, and there are others who believe

* An improper and often dangerous practice, if to any great extent.

this slough to be the cause of pitting, and as being attendant on each pustule that goes through its course of suppuration and pitting—we must hence be led to infer, that if the inflammatory excitement producing this slough be early employed in producing adhesion, the formation of the slough, and consequently future suppuration and ulceration will be prevented. But Mr. Hunter seems to consider, that the formation of the slough was not so much the effect of intensity and degree as the peculiar kind of inflammation—in reply we must observe, that peculiarity of inflammation is a thing we know little about, except from its tendency and effects, that if a healthy adhesive inflammation be produced where an unhealthy tendency to the production of certain known effects existed, then, the existence of that peculiarity of action became of little consequence, being so easily destroyed."

